# Factors influencing national public health governance capacity: based on NCA and fsQCA

**DOI:** 10.3389/fpubh.2025.1628677

**Published:** 2025-09-03

**Authors:** Junyan Li

**Affiliations:** School of Public Administration and Policy, Shanghai University of Finance and Economics, Shanghai, China

**Keywords:** public health governance capacity, configurational pathways, necessary condition analysis, fuzzy-set qualitative comparative analysis, technological, organizational, environmental

## Abstract

**Introduction:**

With the acceleration of globalization, a series of public health emergencies have not only threatened population health but also significantly impacted global economic and political stability, highlighting the urgency for effective public health governance.

**Methods:**

This study employs the TOE framework to examine 149 countries as case studies, utilizing Necessary Condition Analysis (NCA) and fuzzy-set Qualitative Comparative Analysis (fsQCA) to investigate the interactive effects and pathway selections of technological, organizational, and environmental conditions on national public health governance capacity.

**Results:**

The findings reveal the following: (1) No single factor constitutes a necessary condition for high-level public health governance capacity. (2) Technological, organizational, and environmental factors operate in complex “multiple concurrent” configurations, generating diverse pathways that drive national public health governance capacity, illustrating the principle of “different routes leading to the same destination.” (3) Four configurational pathways that enhance national public health governance capacity are identified: configurations jointly explained by technological and organizational factors; configurations collectively determined by technological, organizational, and environmental factors; configurations independently explained by organizational factors; configurations jointly explained by organizational and environmental factors. (4) Representative nations across these four configurational pathways are predominantly high-income countries, with some upper-middle-income countries, while lower-middle-income and low-income countries are notably absent.

**Discussion:**

This research recommends that countries develop targeted strategies to enhance public health governance capacity based on their specific characteristics and resource endowments. Simultaneously, assisting low-income countries in overcoming resource constraints and integrating them into global governance frameworks remains essential.

## 1 Introduction

Since the late 20th century, economic globalization has significantly accelerated cross-border transmission of infectious diseases through enhanced human mobility. This evolution has precipitated the emergence of globalized public health challenges. The 21st century has witnessed escalating frequency of regional and global public health emergencies, including the 2003 SARS outbreak, 2009 H1N1 influenza pandemic, 2014 Ebola epidemic, and the COVID-19 crisis emerging in late 2019. These events typically feature sudden onset, widespread impact, and diverse causes. They threaten both global health security and socioeconomic stability, while also challenging political systems and environmental sustainability. This highlights the need for comprehensive public health governance.

Public health governance refers to the systematic process through which governmental bodies and healthcare institutions employ regulatory interventions, market-based mechanisms, and collaborative strategies to optimize population health outcomes and mitigate large-scale disease burdens. The evolution of socioeconomic systems and technological advancement has precipitated heightened demands for public health governance. First, rising living standards have empowered human populations with both the capacity and motivation to enhance quality of life and health standards, correspondingly elevating societal expectations regarding collective well-being. Concurrently, the advancement of modern scientific technologies has introduced new anthropogenic risks, including widespread food additive contamination, nuclear radiation exposure, and environmental degradation. Consequently, within the contemporary modernization trajectory, there exists an urgent imperative to substantially enhance public health governance capacity, thereby minimizing human vulnerability to emerging health risks. Public health governance capacity constitutes a critical dimension of governmental administrative competence, encompassing the state’s multidimensional capability to deliver health products, coordinate service delivery, and maintain salubrious environments.

Scholars have conducted extensive and constructive research investigating public health governance, including healthcare system reform (1), institutional relationships between governmental entities, healthcare providers and citizens (2), digital governance strategies (3), and artificial intelligence applications (4). Existing scholarship has predominantly examined the governmental responsibilities and roles in public health emergency response through focused case-specific analyses (5). Utilizing an analytical framework that systematically deconstructs “current public health governance status—governmental capacity limitations—constraining factors influencing governmental intervention” (6), researchers have explored government emergency management capabilities when confronting public health emergencies, and proposed normative optimization strategies. Currently, most research focuses on summarizing public health governance practical experiences and conducting qualitative descriptive analyses, lacking in-depth empirical research. While some studies have developed governmental governance performance evaluation indicators and conducted empirical research (7), their limited research perspectives and analytical frameworks make it difficult to gain a profound understanding of public health governance’s essence and mechanisms. This limitation ultimately obscures potential pathways for enhancing public health governance capabilities.

Current research on public health governance remains at the preliminary stage of theoretical construction. Multiple distinct research perspectives, analytical levels, and methodological approaches coexist, with primary focus on examining independent effects of technological or organizational factors. This approach limits understanding of the coordinated matching effects among different factors that underlie differences in public health governance capacity. The impacts of diverse factors on public health governance capacity are not independent, as these elements generate combined effects through coordinated interactions. Consequently, employing a “configurational perspective” in research facilitates deeper comprehension of the complex mechanisms underlying governance variations across nations (8). This study empirically explores the influencing factors and improvement pathways of public health governance capacity by utilizing the “configurational perspective.” Specifically, the study aims to address the following questions: What conditions are configured in a “similar outcomes through different paths” manner to enhance public health governance capacity? Which conditions are most critical for improving public health governance capacity? What matching and substitution relationships exist among them? This study constructs a Technology-Organization-Environment (TOE) research framework, and using 148 countries as cases, employs Necessary Condition Analysis (NCA) and fuzzy-set Qualitative Comparative Analysis (fsQCA) to examine the condition configurations that drive high public health governance capacity. This research broadens the perspective on public health governance studies and deepens our understanding of the pathways and mechanisms that drive it.

This study offers several potential marginal contributions: First, this research transcends the limitations of traditional linear analysis from a configurational perspective to reveal the complex causal mechanisms underlying public health governance capacity across different nations. By identifying multiple synergistic pathways among technological, organizational, and environmental factors, it addresses the inadequacies of existing literature that focuses on independent effects of singular factors. Second, by combining NCA and fsQCA, this study both validates the necessity strength of conditions and uncovers sufficiency pathways through multi-condition coupling. These approaches provide a new paradigm for understanding the differentiated performance of governance capacities and offer specific references for policy formulation across countries. Third, this research constructs a multi-level analytical framework for public health governance by integrating resource-based view, institutional theory, and health determinants theory. It offers both theoretical support and practical guidance for improving public health policies and promoting global health equity.

The remainder of this paper is structured as follows: Section 2 provides a comprehensive review of the relevant literature. Section 3 introduces the research framework from technological, organizational, and environmental perspectives. Section 4 describes the research design, including research methods, indicator selection, data sources, and calibration. Section 5 presents the research findings, encompassing necessary condition analysis, configuration analysis and robustness tests. Section 6 summarizes the conclusions, offers policy implications, and presents limitations and future research directions.

## 2 Literature review

Reviewing existing research, scholars have conducted detailed research into critical dimensions of public health governance, including its influencing factors, practices and challenges, achieving substantial scholarly outcomes.

### 2.1 Public health governance influencing factors

Public health governance, as a critical domain of social governance, is intricately shaped by multiple interacting factors. From a governance structural perspective, World Bank research demonstrates that effective health governance systems must encompass key institutional elements such as transparency, accountability, and participatory decision-making mechanisms. These institutional arrangements significantly influence health fiscal expenditure efficacy through mediating variables including political stability, rule of law, and governmental effectiveness (9). Beaglehole et al. (10), in their earlier work, emphasized that public health interventions must strategically embed policies within supportive systemic frameworks through collective action to simultaneously achieve health equity and sustainable improvement. In practice, governance structures require information systems for support. McQuide et al. (11) note that as global development priorities have shifted from the Millennium Development Goals (MDGs) to the Sustainable Development Goals (SDGs), health human resources information systems (HRHIS) in low- and middle-income countries (LMICs) have evolved from initially fragmented data collection tools into core infrastructure supporting health workforce planning. It has also become a key component of effective governance. This evolution reflects how governance mechanisms enhance decision quality through data integration, especially in resource-constrained settings.

Public health inherently embodies cultural dimensions. Beyond biological mechanisms, disease control encompasses sociobehavioral, political, and economic determinants (12). Medical technologies, economic growth (nutritional improvements), public health measures and educational expansion constitute the knowledge framework for improving human health. Historical evidence demonstrates that modern medical advancements since the 19th century, including novel prevention methods, vaccines, and antimicrobial therapies have successfully controlled persistent infectious diseases that long plagued humanity (13). Post-1950s research has established education’s critical role in health promotion. Education exerts both direct effects through health knowledge dissemination and indirect impacts via socioeconomic mediators like income, employment, and living conditions (14). A multinational panel study by Lutz et al. (15) confirms that educational attainment surpasses income growth as the predominant driver of life expectancy extension and child mortality reduction globally. Research by Vaupel et al. (16) demonstrates that the extension of human lifespan primarily stems from environmental and behavioral modifications, which mitigate health risks through environmental interventions, apply medical technologies to reduce disease mortality, and enhance geriatric health via nutritional improvements and early-life disease prevention. Yet in recent years, the intertwined relationship between environment and public health has become a core issue in global governance (17). Climate change-induced extreme weather and ecological disruptions amplify health risks by altering pathogen transmission routes and intensifying food security crises. However, existing governance tools lack systematic intervention in these upstream drivers (18, 19). In the increasingly diverse 21st century, health is increasingly regarded as a fundamental human security and an essential right. Global health governance mechanisms must prioritize fundamental health determinants, focusing on proactively controlling and preventing disease transmission from its source (20). Meanwhile, the synergy between technological innovation and governance mechanisms has emerged as a key factor influencing public health effectiveness. Research by Wu et al. (21) shows that the role of technology in public health is not isolated. Instead, it requires effective governance mechanisms to enable the full chain of “technology identification-integration-application.” This theoretical framework explains how technology translates into practical governance effectiveness. At the individual level, Leporatti and Montefiori (22) reveal that individuals with low digital skills face a higher probability of worsening health. Conversely, living in highly digitized countries can reduce health risks by 1–2.7%. This indicates that digital inclusivity has become an indispensable consideration in modern public health governance, especially amid public health emergencies.

From a resource allocation perspective, scholars increasingly agree on the positive correlation between financial investment and long-term governance effectiveness. Bloom et al. (23) demonstrated through cross-national comparisons that, under comparable income levels, public health investment intensity significantly correlates with life expectancy. This conclusion finds further microeconomic validation. Brown (24) empirical research on California revealed that public health investments can generate economic returns ranging from 67 to 88 times the initial investment. However, investment effectiveness is not solely determined by financial scale. When increasing health investments, LMICs must simultaneously strengthen anti-corruption oversight, enhance policy enforcement, and improve monitoring mechanisms. Otherwise, they risk falling into the “high input, low output” dilemma (25). Consequently, increasing public health financial investment represents a judicious governance strategy, but it requires supporting institutional optimization. Developing countries consistently confront resource constraint challenges, a context where Bennett (26) discovered institutional innovation can effectively compensate for resource limitations. Case studies from Kerala (India), Tanzania, Sudan, and Venezuela substantiate how innovative health governance can achieve comprehensive primary healthcare coverage. Resource constraints are not limited to financial shortages but also encompass emerging demographic structural challenges. Goodman-Palmer et al. (27) found that the aging wave is placing unprecedented pressure on healthcare systems in LMICs. While economic growth has driven increases in overall health investments, services for older adults in these countries remain heavily reliant on fragile primary healthcare systems, rendering resource allocation increasingly complex. Moreover, the prevalence of non-communicable diseases (NCDs) has emerged as a critical underlying factor affecting public health governance effectiveness. Elkomy and Jackson (28) highlight that NCDs account for 70% of global mortality. These diseases not only directly threaten population health but also undermine national healthcare system resilience through long-term medical burdens. During the COVID-19 pandemic, patients with NCDs, due to low immunity, were more likely to develop severe symptoms. This demonstrates that the prevention and control level of NCDs directly influences a nation’s capacity to respond to public health emergencies, constituting a fundamental and unavoidable impact factor in public health governance.

### 2.2 Public health governance practices

The international community’s endeavors in public health governance have been intrinsically intertwined with the historical lessons of major infectious diseases. As globalization accelerates, diverse elements circulate rapidly across global networks, particularly via advanced transportation technologies such as aviation, cold-chain logistics, and high-speed rail. This rapid circulation has dramatically expanded the potential transmission scope and velocity of pandemics, rendering traditional containment strategies increasingly obsolete (29). Concurrently, global public health surveillance systems remain fragmented, critically undermining both emergency response capabilities and systematic coordination mechanisms (30). Consequently, a multi-tiered health governance system gradually emerged. By the end of 2004, the European Commission established the European Centre for Disease Prevention and Control (ECDC), mandated with comprehensive functions including disease surveillance, information gathering, early warning systems, timely response mechanisms, scientific advisory services, microbiological monitoring, public health workforce training and comprehensive risk assessment of emerging infectious diseases (31). The 2005 International Health Regulations underwent significant revisions, aimed at enhancing the World Health Organization’s governance capacity in addressing increasingly prominent infectious disease challenges. In 2007, the Security and Prosperity Partnership (SPP) of North American leaders formulated the North American Plan for Animal and Pandemic Influenza (NAPAPI). Following the 2009 H1N1 outbreak, the North American Leaders Summit (NALS) succeeded the SPP, implementing an enhanced NAPAPI framework. The plan’s key priorities included establishing a collaborative communication platform for coordinated regional responses, preventing pandemic entry, mitigating socioeconomic disruptions and minimizing infection and mortality rates. Beyond the European Union and North American regions, other international bodies progressively developed regional health governance frameworks. The Association of Southeast Asian Nations (ASEAN) and the Southern African Development Community established their respective collaborative mechanisms in 2003 and 2007 (32, 33). China contributed uniquely to international health development through medical technology exchanges and supporting healthcare professional training in developing countries (34). The “Shared World, Shared Health” strategic framework proposed coordinated global resource control for influenza viruses and increased investment in disease surveillance (35). The BRICS countries committed to advancing Universal Health Coverage (UHC), exploring a novel global health agenda distinct from Western approaches. Their strategy primarily focused on providing financial support to international health organizations like World Health Organization (WHO) and facilitating technological transfers to developing countries (36).

The COVID-19 pandemic has driven innovation and transformation in governance mechanisms in the post-pandemic era. This crisis has prompted many countries to reassess their health systems. It has not only increased investment in health infrastructure but also highlighted the critical role of strong leadership, clear accountability, rapid social mobilization, high public trust, and effective communication in response efforts (37). At the regional level, the European Union has advanced the European Health Union initiative. By strengthening cross-border solidarity and trust mechanisms, and increasing budgets for healthcare, research and development, and crisis management, it has enhanced public health emergency response capabilities. This has made it a model for regional governance transformation (38). At the national level, India has strengthened its pandemic emergency response capabilities by establishing rapid response teams that integrate multidisciplinary experts in public health, epidemiology, and respiratory medicine. Additionally, in rural areas, monthly clinical case conferences have increased the rate of standardized treatment for childhood pneumonia by 37.5 percentage points, offering a valuable reference for primary public health practices (39, 40). Italy, through its National Recovery and Resilience Plan, has invested in community health centers and family doctor teams. This has enabled primary-level services to take on more preventive and diagnostic tasks, balancing routine care with emergency response (41). East Asian countries, such as South Korea and Singapore, having experienced outbreaks like SARS, developed rapid testing, contact tracing, and stratified response systems. These approaches further demonstrates that integrating public health into infrastructure investments is key to enhancing resilience (42).

Digital technologies have accelerated their integration into public health governance during the COVID-19 pandemic (43, 44). Historically, healthcare systems in LMICs have struggled with persistent challenges of insufficient transparency and weak accountability mechanisms. The emergence of digital technologies offers a novel pathway to address these limitations (45). Artificial Intelligence (AI) is regarded by global health organizations as a potential tool to address healthcare resource gaps and promote health equity in LMICs. It demonstrates promise in enhancing service accessibility and optimizing disease screening (46). However, AI confronts significant structural risks. Models trained predominantly on high-income countries’ data may generate diagnostic biases. The absence of robust evaluation standards and regulatory frameworks could allow substandard products to enter the market. In regions with gender inequalities, AI technologies might further compromise women’s healthcare safety (47, 48). Adaptation challenges among older adults or individuals with low digital literacy may exacerbate the “digital divide” in health services. Ethical concerns surrounding data security and privacy protection could further undermine public trust (49). Concurrently, the pandemic has catalyzed innovations in governance concepts and practices. The “One Health” approach transcends disciplinary boundaries, situating health within a socio-ecological systems framework. It critically examines how drivers such as climate change and biodiversity loss influence infectious disease outbreaks (50).

Public health governance systems inherently exhibit fundamental structural contradictions. Constrained by narrow national interests, international responses to infectious diseases predominantly reflect the priorities of affluent nations rather than addressing the critical needs of economically disadvantaged countries (51). Developed countries have wielded excessive influence over global health agendas, driven largely by self-protection strategies that prioritize preventing the spread of diseases from developing regions to their own. This approach systematically neglects alleviating disease burdens in impoverished countries and fails to provide the essential technical assistance and resources necessary for improvements in public health infrastructures (52). Post-COVID-19, these structural contradictions have become more pronounced. The Conference of Parties (CoP) to WHO CA + is open only to countries that have ratified the treaty. This may exclude non-ratifying nations from core governance processes, undermining the comprehensiveness of global coordination (53). Actions such as the United States’ withdrawal from some global health programs and reduced support for the WHO reflect a tendency among some countries to prioritize their own interests in global health governance (54). Indonesia’s new health legislation, due to insufficient public engagement and neglect of critical issues, has low policy acceptance. This highlights a misalignment between priorities in governance processes and public needs (55). During the COVID-19 pandemic, shortages of key diagnostic equipment like pulse oximeters exposed the vulnerability of traditional supply chains in emergency responses (56). Moreover, countries with robust technological infrastructure can more effectively leverage the dividends of digital technology. In contrast, low-income countries with weak digital infrastructure and insufficient knowledge spillover effects, struggle to implement telemedicine or online collaboration, hindering the execution of prevention and control measures. Fundamentally, this reflects unequal global health resource distribution and a governance system that fails to adequately address the needs of vulnerable countries (57).

### 2.3 Public health governance challenges

The fundamental challenge confronting global public health governance lies in the profound disparities in public health investment and developmental capacities across nations. Existing scholarship illuminates the disadvantages experienced by developing countries within the global public health governance framework, while revealing critical systemic deficiencies in capacity building, particularly the urgent need for substantial financial and technological support (58). Economic divergence imposes significant fiscal and resource constraints on public health infrastructure in developing countries. According to WHO data, while developing countries incrementally increased their global health expenditure share from 13 to 19% between 2000 and 2017, high-income nations still dominated with approximately 80% of total investment. The economic chasm is starkly evident in per capita medical expenditure. In 2017, low-income countries averaged merely $41 per person, compared to $2,937 in high-income nations—a seventy-fold difference. Primary health care (PHC) is a core element of achieving high-quality universal health coverage (UHC), yet it has not received the attention it deserves in the policy agendas and practices of LMICs (59). Annual per capita spending on PHC in LMICs is only $15–$60, far below the $97 needed to achieve 80% service coverage. This funding gap directly results in insufficient coverage of basic services (60). Under these economic constraints, many developing countries not only lack sufficient resources to establish robust health systems but have also exhausted most of their limited resources addressing existing health challenges and meeting basic population health needs (61). The Democratic Republic of the Congo (DRC) serves as a typical example. Economic hardship directly limits public health investment, compounded by poor coordination in provincial governments’ pandemic response, chronic underfunding, and corruption in public procurement. This has led to recurring outbreaks of infectious diseases such as cholera (62, 63). This underscores the urgent need for comprehensive global health governance reforms and equitable resource allocation.

While the WHO and high-income member states provide technical and financial assistance to nations in need, these resources remain inherently constrained (64). The WHO has been unable to address its persistent funding and staffing deficits, thereby limiting its capacity to comprehensively tackle global health challenges and exacerbating difficulties in developing nations. By the late 2000s, the organization’s financial constraints had become increasingly evident. As of early 2021, WHO maintained merely 200 full-time personnel dedicated to implementing the International Health Regulations (IHR), with associated staffing costs totaling approximately $42 million—a resource allocation proving insufficient to address contemporary global health threats (65, 66). Scholars have further noted that the global public health system established by the IHR largely relies on member states’ ability to build response systems, yet significant implementation gaps persist in practice (67). Few countries have explicitly designated their assistance to fund projects building IHR-mandated core capacities. Numerous studies indicate that public-private partnerships (PPPs) can address certain public health challenges in the world’s poorest nations at reduced costs (68). However, these collaborative mechanisms also exert detrimental impacts on global health governance. PPPs disproportionately prioritize pharmaceutical and vaccine development while neglecting the vulnerabilities in health infrastructure and limited drug and vaccine distribution capacities endemic to low-income developing countries. More critically, such partnerships frequently impose conditionalities during aid program implementation. These requirements not only burden recipient nations’ healthcare infrastructures but also hinder medical assistance from reaching impoverished populations, thereby exacerbating health inequities (69, 70). Additionally, PPPs in LMICs frequently encounter issues such as weak regulatory frameworks, uneven private sector capacities, and limited public trust. Successful cases, however, typically feature government-led regulatory coordination, flexible contract design, and adaptability to local needs (71). From the perspective of financial allocation, there is a disproportionate allocation of limited resources toward vertical disease-specific programs, particularly targeting HIV/AIDS, malaria and tuberculosis, rather than core capacity development (61). Scholars contend that this preferential funding approach creates inefficiencies by diverting resources from comprehensive public health system development and sustaining an unsustainable model that fails to achieve long-term epidemic control objectives (72).

The COVID-19 pandemic profoundly exposed structural inequalities in governance and generated unique challenges in the post-pandemic era (42). On one hand, vaccine distribution was severely unequal, with many African countries achieving vaccination rates below 10% while high-income countries stockpiled vaccines. Moreover, trade protectionism led to export restrictions on personal protective equipment, ventilators, and other critical medical supplies, further undermining the foundations of global collaboration (73–75). On the other hand, marginalized populations (low-income groups, ethnic minorities, migrants) experienced disproportionate infection rates, mortality, and economic losses. These disparities stem from long-standing structural barriers, including unequal medical resource allocation, high prevalence of underlying conditions due to poverty, and elevated occupational exposure risks. Such problems were dramatically amplified during the pandemic (76). For instance, residents of Bangladeshi slums were forced to forgo non-emergency medical care due to lost daily wages, while Nigerian rural families could not access medical facilities because of transportation disruptions. This reflects the vulnerability of social and health systems in LMICs’ response to emergencies (77). The pandemic caused the first widespread decline in the Global Sustainable Development Goals (SDGs) index. Low-income countries experienced the most severe impact, while high-income countries, with more resilient health systems, were comparatively less affected, triggering a Matthew effect (78). Simultaneously, the pandemic fully exposed weaknesses in cross-governmental collaboration and multi-sectoral coordination. In LMICs, widespread institutional fragmentation and lack of coordination mechanisms—such as hierarchical barriers within health systems blocking information flow, cross-sectoral coordination bodies reduced to “symbolic entities” due to lack of budgetary autonomy and decision-making authority, and frequent rotations in civil service systems disrupting continuity of collaboration—all constrained emergency response efficiency (39, 79). These challenges collectively point to a core contradiction: existing systems overemphasize “controllability” but struggle to address recurring “irregularities” during the pandemic, such as the unpredictability of viral mutations and public resistance to prevention measures. Balancing structural transformation with managing uncertainty has become a key proposition for global health governance in the post-pandemic era (80).

## 3 Model construction

**Figure 1:**
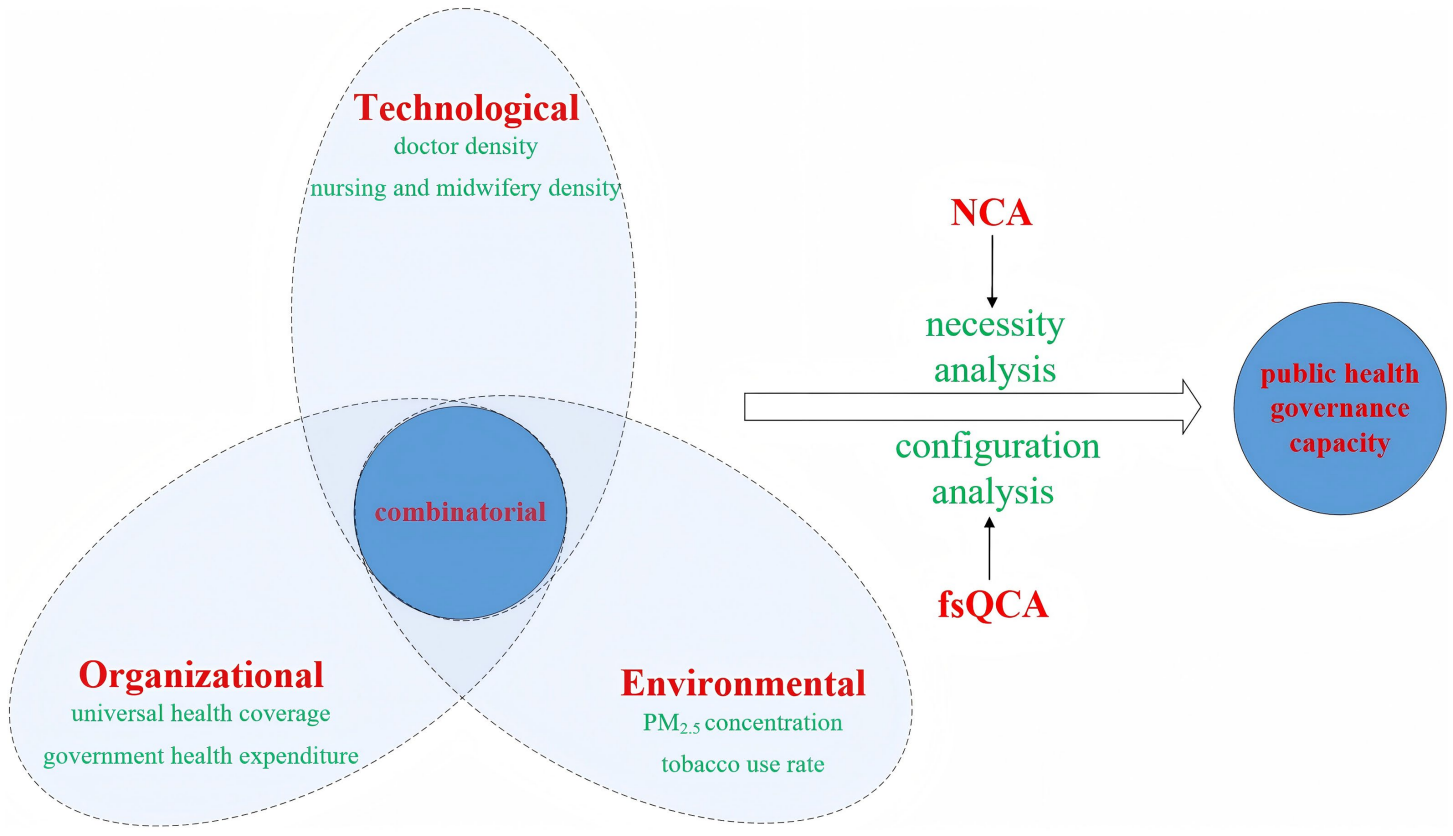
Analytical framework.

While some research has begun to address the differentiated pathways of governmental governance (81), exemplary frameworks or reference models for explaining these governance variations remain scarce. Existing literature exhibits several critical limitations: First, despite providing rich explanations for public health governance, current research fails to offer robust theoretical support for identifying differentiated governance pathways. Second, the enhancement of public health governance capacity involves interdependent rather than independent conditions. Existing literature’s assumption of uniform symmetric relationships between independent and dependent variables has constrained the selection of pathways for improving public health governance capacity. Third, in real-world contexts, public health governance involves complex logical relationships between configuration patterns and outcomes—specifically, understanding which condition configurations trigger or inhibit specific result variables. Notably, the conditions facilitating high public health governance capacity may fundamentally differ from those contributing to low capacity. Existing research has insufficiently addressed the causal complexity inherent in public health governance capabilities. To address these limitations, this study introduces fsQCA to explore the interactive effects of technological, organizational, and environmental factors on public health governance capacity. By leveraging the TOE analytical framework, the research constructs a model framework elucidating the determinants of public health governance capacity, as illustrated in Figure 1.

### 3.1 Technological condition

The institutional environment provides fundamental constraints that govern organizational conduct, making the comprehension of embedded institutional contexts a prerequisite for analyzing organizational behavior (82). Grounded in the Resource-Based View (RBV) theory, organizational strategic choices are inherently limited by their resource endowments (83). Initially emerging from strategic management research (84), RBV posits that organizations function as resource integrators (85). Barney (86) systematically expanded this theory by identifying four defining characteristics—value, rarity, non-substitutability and imperfect imitability—that establish specific resources as core sources of sustainable competitive advantage. While RBV was principally applied to private sector studies in its early development (87), its analytical framework has since been extended to public administration contexts (88, 89). This theoretical expansion proves particularly salient in professionalized sectors such as healthcare, where specialized competencies constitute vital organizational capital (90).

In public health governance, human capital constitutes one of the most strategically vital resources within institutional environments. Paoloni et al. (91) identifies human capital as an organization’s accumulated tacit resource whose non-replicable nature forms the cornerstone of competitive advantage. This logic proves particularly evident in healthcare systems, where clinical human capital scarcity directly impacts public health responsiveness and service quality. Professionally trained medical personnel, for instance, not only possess technical expertise but also develop unique service capabilities through experiential learning, thereby enhancing health institutions’ resilience during emergencies. In essence, the accessibility and quality of human capital shape public health governance efficacy by strengthening organizational core competencies (92). Thus, within resource-constrained institutional settings, human capital serves dual functions as both foundational input and long-term competitive driver for public health systems (93). The doctor density and the nursing and midwifery density reflect a nation’s healthcare system’s technological capability and professional talent reserves (94). Higher density rates not only expand coverage of routine medical services but also enable more rapid and precise responses to public health events, thereby strengthening overall governance capacity (95–97). These two indicators collectively form the technological foundation of health systems and significantly influence a country’s capabilities in disease prevention, surveillance and response (98, 99).

### 3.2 Organizational condition

Within the organizational dimension, Scott’s (100) institutional theory identifies the regulative pillar as constituting the foundational dimension of institutional frameworks. This pillar imposes structural constraints on organizational behavior through rule-making, monitoring and sanctioning mechanisms. Particularly applicable to public health governance, this framework provides critical insights into how mandatory normative systems enhance institutional effectiveness through coercive compliance mechanisms.

Universal Health Coverage (UHC), a core Sustainable Development Goal, fundamentally embodies the regulatory characteristics of institutional systems (101). As the key metric for assessing accessibility and equity in public health services, UHC requires not only breakthroughs in population coverage but also institutional arrangements addressing health rights protection, resource allocation mechanisms and social equity adjustments (102). The institutionalization of UHC necessitates governments to establish inclusive healthcare security systems, such as legally mandating basic medical services for all citizens. This institutional design clarifies governmental responsibilities (e.g., financing and service delivery) while transforming health rights from ethical claims into legal entitlements through legislative enforcement. Japan’s National Health Insurance Law demonstrates this approach through compulsory enrollment and tiered payment systems, achieving 99% insurance coverage. This shifts healthcare resource allocation from “market selection” to “institutional compulsion,” reducing health inequities caused by economic disparities. The Netherlands employs a hybrid healthcare system based on mandatory health insurance to achieve universal coverage. Ireland, Spain, Italy and Belgium all implement universal healthcare systems. UHC evaluates both the breadth and depth of essential health service provision. Higher UHC index indicate stronger institutionalization of basic medical services, catastrophic disease protection and health rights for vulnerable populations. These organizational-level institutional designs effectively bridge health inequities and enhance public health governance resilience (103).

The proportion of government health expenditure ensures baseline governance capacity through budgetary system constraints (104–106). As mandated by the WHO’s Alma-Ata Declaration target of “health for all,” the global minimum threshold requires national health expenditure to constitute at least 5% of GDP. This institutional rigidity compels governments to prioritize public health resource allocation, insulating investments from political cycles or economic fluctuations. The government health expenditure serves as a quantifiable institutional commitment indicator. Higher expenditure necessitates systematic deployment of budgetary mechanisms (e.g., UK’s NHS) and fiscal instruments (e.g., Thailand’s sin taxes financing the 30 Baht Program) to ensure sustained resource flow into public health systems. This institutionalized allocation reflects governmental prioritization of health resource distribution (107, 108). Moreover, resource dependence theory emphasizes organizations’ dependence on critical resources. Government health expenditure, as a core resource input, directly determines the public health system’s infrastructure construction, talent cultivation, technological innovation and emergency response capabilities. Stable and predictable funding guarantees enable public health institutions to implement long-term health interventions and systemic capacity building, rather than merely responding to emergent events (109, 110).

### 3.3 Environmental condition

The Social Determinants of Health (SDOH) theory provides essential guidance for formulating public health policies and interventions (111). Social environments and associated environmental hazards/exposures impact health through diverse mechanisms. When these circumstances demonstrably affect health outcomes, they are systematically defined as SDOH. These determinants should be recognized as critical environmental factors for improving public health systems and medical services, given their extensive influence on population health, functional capacity, and quality of life (112). Among these, environmental pollution risks and behavioral risks reflect, from different dimensions, the public health environmental pressures faced by a nation.

Urban PM_2.5_ concentrations reflect the level of environmental pollution risks faced by a society (113, 114). PM_2.5_ governance requires deep integration of technology empowerment and institutional innovation. Technologically, real-time air quality monitoring networks (e.g., China’s “Blue Sky Defense Campaign” deploying over 1,700 national air quality monitoring stations) rely on IoT and big data technologies, demonstrating the technological absorption capacity of governance systems (115, 116). Institutionally, haze crisis response requires joint action mechanisms involving environmental protection, health and transportation departments. For example, when New Delhi’s PM_2.5_ levels exceeded 500ug/m^3^ in 2019, the government implemented tiered responses including vehicle restrictions, factory shutdowns and school closures. However, unclear departmental responsibilities caused implementation delays, exposing weaknesses in collaborative governance (117). Under such high-pressure scenarios, PM_2.5_ concentrations become a “stress indicator” testing governance system resilience. This demonstrates that heavily polluted areas require public health systems with stronger environmental health monitoring and response capabilities. Furthermore, air pollution control is a quintessential cross-sector collaboration issue requiring coordinated participation from environmental, health, industrial and transportation departments. Nations demonstrating effective air pollution control capabilities are characterized by well-established interdepartmental coordination mechanisms, comprehensive monitoring and early warning systems and effective intervention measures. These competencies collectively constitute critical components of public health governance capacity.

Tobacco use rates serve as behavioral risk indicators that measure the prevalence of tobacco-related health risks, functioning as critical metrics for assessing chronic disease burdens and reflecting societal health behavior patterns and public health education effectiveness (118–120). High tobacco consumption directly increases the incidence of cardiovascular diseases and cancers while exacerbating healthcare resource demands, creating sustained pressure on public health systems. Simultaneously, tobacco control represents a core component of modern public health governance, requiring multi-sector collaboration, legal regulation and policy implementation (121). Over the past decade, tobacco control has achieved unprecedented prominence in global health, exemplified by the widespread ratification of the WHO Framework Convention on Tobacco Control (FCTC)—the first international public health treaty. The FCTC is recognized as both a governance tool and a model for combating non-communicable diseases. Countries demonstrating effective tobacco control typically possess stronger public health legislation, more efficient health education systems and enhanced policy enforcement capabilities-fundamental elements of public health governance capacity.

## 4 Research design

### 4.1 Research methods

Necessary and sufficient causal relationships represent two emerging causal explanations in research. Necessary causation indicates that a particular outcome cannot occur without the presence of a specific antecedent condition. In contrast, sufficient causation refers to when an antecedent factor (or combination of factors) is adequate to produce the outcome (122). To better analyze both necessary and sufficient causal relationships in this study, the research first employed NCA, a novel methodological approach (123). While fsQCA can identify necessary relationships, it only qualitatively states whether “a condition is necessary or unnecessary for a result” without quantifying the degree of necessity—specifically, “to what extent a condition becomes necessary for a result.” This integration of NCA with fsQCA offers particular value when working with fuzzy sets, as these involve not just binary “yes” or “no” determinations but detailed membership scores, thereby enhancing the analytical power of the combined approach (124).

Departing from traditional statistical approaches rooted in binary variable relationships, this study employs fsQCA, a method grounded in set theory, to investigate the complex, multifaceted mechanisms underlying public health governance capacity through a configurational perspective. This methodological choice is motivated by two primary considerations. Primarily, conventional statistical analyses examining the independent or interactive effects of factors such as doctor density, universal health coverage, and government health expenditure prove insufficient for uncovering the nuanced pathways to enhanced public health governance capacity. Unlike traditional analytical techniques, QCA posits that causal conditions are inherently interdependent, with their diverse combinations constituting multiple conjunctural causal relationships (125). This approach facilitates a more comprehensive understanding of the differentiated driving mechanisms of public health governance across global contexts. Consequently, QCA provides a more robust methodological framework for investigating the intricate mechanisms of public health governance capacity by prioritizing holistic relational dynamics.

Moreover, the diversity of pathways for enhancing public health governance capacity across nations suggests the potential existence of multiple “equifinal” causal chains converging toward the same outcome. While traditional statistical approaches can comprehensively analyze factors influencing public health governance capacity through mediating and moderating variables, they predominantly explain outcome variations through substitution or cumulative relationships between variables, rather than capturing their fully equivalent mechanisms. In contrast, the QCA method is capable of identifying distinct configurations of antecedent conditions that are fully equivalent yet non-conflicting in explaining the outcome (126). Compared to conventional statistical methods, QCA is demonstrably more suitable for investigating the diverse pathways of national public health governance capacity enhancement.

Since 2000, scholarly attention to QCA in empirical research has demonstrated a marked upward trajectory, spanning diverse disciplines including political science, sociology, management, and international relations. The methodological landscape of QCA encompasses three fundamental variants: crisp-set QCA (csQCA), fuzzy-set QCA (fsQCA), and multi-value QCA (mvQCA). In contrast to csQCA and mvQCA, which are primarily confined to categorical analyses, fsQCA offers superior analytical flexibility by enabling the examination of nuanced degree variations and partial set memberships. Consequently, fsQCA has garnered widespread adoption in contemporary empirical research.

### 4.2 Data and calibration

#### 4.2.1 Data sources

The research data is sourced from the World Health Statistics 2023. This report, an annual compilation by the WHO, provides the latest data on health indicators and health-related metrics across its 194 member states, encompassing over 50 health-related indicators from the Sustainable Development Goals and WHO monitoring frameworks. Guided by the public health governance theoretical framework, and after excluding samples with missing data, this study selected data from 149 countries as the research sample.

#### 4.2.2 Outcome variable

The World Health Statistics 2023 reports 15 core capacities required by the International Health Regulations (IHR), spanning critical public health domains including surveillance, emergency response, laboratory capabilities, risk communication and border health measures etc. These capacities are designed to empower nations to effectively prevent, detect and respond to public health events of international significance. Consequently, the average of these 15 IHR core capacity scores emerges as a comprehensive metric for evaluating national public health governance capability.

#### 4.2.3 Antecedent variables

Technological conditions: Encompassing doctor density, nursing and midwifery density. Doctor density is measured by the number of medical doctors per 10,000 population. Nursing and midwifery density is evaluated by the number of nursing and midwifery personnel per 10,000 population.

Organizational conditions: Comprising universal health coverage and government health expenditure. Government health expenditure refers to the percentage of domestic general government health expenditure within general government expenditure.

Environmental conditions: Including PM_2.5_ concentration and tobacco use rate. PM_2.5_ concentration represents the annual mean level of fine particulate matter (PM_2.5_) in urban areas, measured in micrograms per cubic meter (μg/m^3^). Tobacco use rate represents the age-standardized prevalence of tobacco use among persons 15 years and older.

#### 4.2.4 Calibration

In fsQCA, calibration refers to the process of assigning set membership to cases. Specifically, researchers must calibrate variables into sets based on existing theoretical knowledge and case contexts. Calibrated set membership scores range between 0 and 1. To calibrate variables within this interval, researchers select calibration anchor points (full membership, crossover point, and full non-membership) by examining the actual distribution of variable values and considering case-specific contexts. Drawing on existing research, this study employs direct calibration (Ragin and Fiss (100)) to convert data into fuzzy-set membership scores, grounded in theoretical and empirical knowledge. The calibration process involved three key steps: (1) calculating quantiles at 0.75, 0.50, and 0.25 for each variable; (2) establishing 0.75 quantile as the full membership threshold, 0.50 as the crossover point, and 0.25 as the full non-membership threshold; (3) Cases with a calibrated membership score of 0.5 for outcomes or antecedents are automatically removed by the software, which can compromise result accuracy. Following existing research, I add a constant of 0.001 to all data with a calibrated membership score of 0.5. Notably, PM_2.5_ concentration and tobacco use rate are negative indicators, meaning that higher values indicate poorer environmental conditions. The following formula was used to convert them into positive indicators: Transformed value = Maximum value of the original indicator in the sample—Original value. This transformation ensures the directional consistency of all antecedent variables, with higher transformed values corresponding to more favorable conditions (e.g., lower PM_2.5_ concentration or lower tobacco use rate), thereby aligning their logic with positive indicators. Calibration was performed after this transformation. The calibration results and descriptive statistics for all outcome and antecedent variables are presented in Table 1.

**Table 1:** The calibration and descriptive statistics.

Variables		Thresholds for the calibration process			Descriptive statistics			
		Full membership	Cross-over point	Full non-membership	Max	Min	Mean	Std. dev
Outcome variable	Public health governance capacity	81	68	54	100	36	67.7	16.7
Technological variables	Doctor density	34.7	19.9	3.9	84.3	0.3	21.2	18.3
	Nursing and midwifery density	71.9	35	14.5	223.2	1.9	49.9	45.9
Organizational variables	Universal health coverage	80	71	53	91	29	66.7	16.1
	Government health expenditure	14	10.7	7.4	25.2	2.4	11.0	4.82
Environmental variables	PM_2.5_ concentration	63.3	56.8	42.6	70	0	51.6	15.3
	Tobacco use rate	32.7	23.9	18.6	40.6	0	24.6	9.41

Calibration thresholds are determined based on quantiles: full membership corresponds to the 0.75 quantile, cross-over point to the 0.50 quantile, and full non-membership to the 0.25 quantile. PM_2.5_ concentration and tobacco use rate are negative indicators and have been converted into positive indicators.

## 5 Results and analysis

### 5.1 Single-factor necessity analysis

#### 5.1.1 NCA single-factor necessity analysis

**Figure 2:**
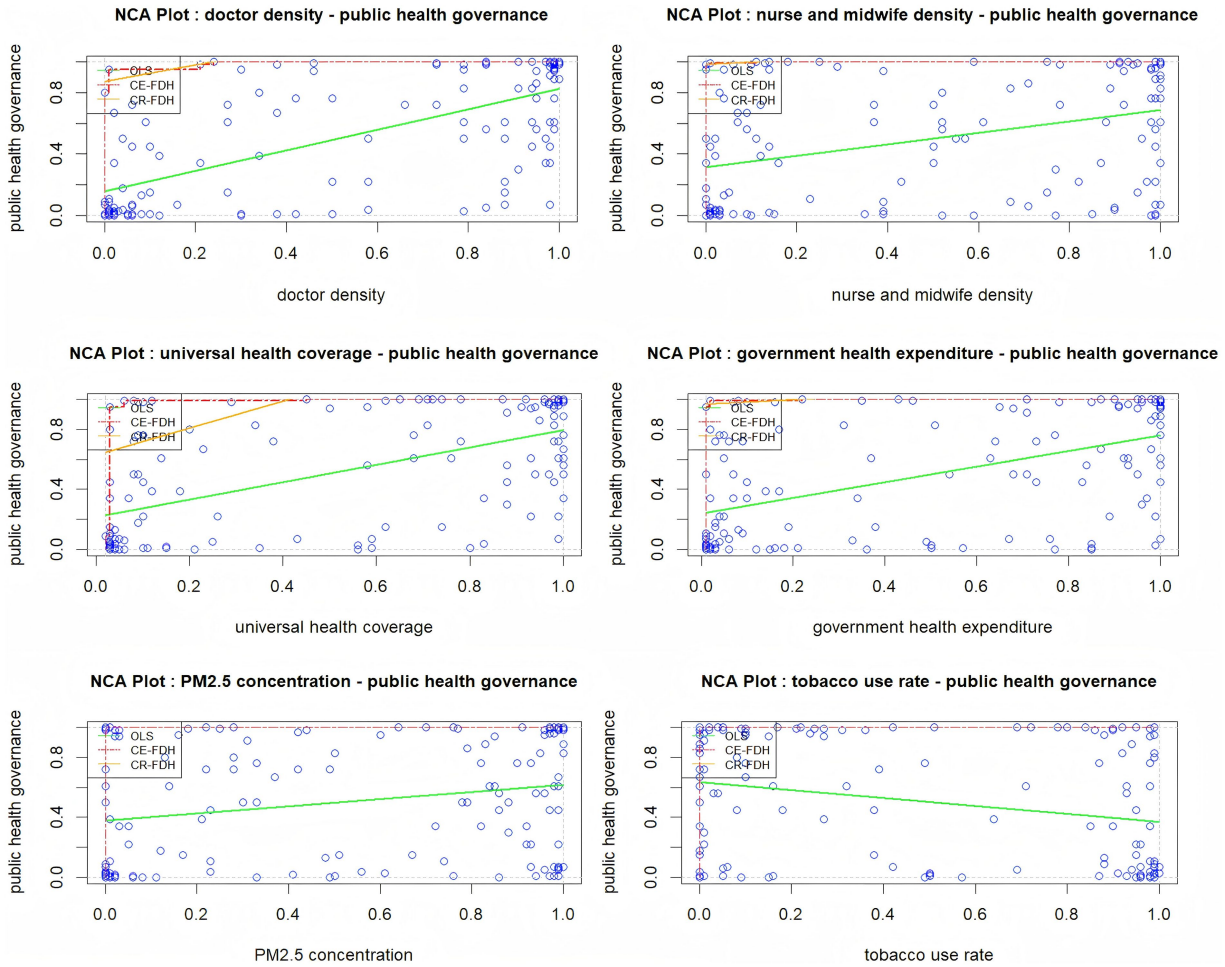
Scatter plot of antecedent condition.

Prior to conducting configuration analysis, it is crucial to individually examine the necessity of each condition. The NCA enables the assessment of whether and to what extent a specific condition serves as a necessary condition for an outcome. R 4.4.3 software is used to conduct NCA analysis. The NCA analysis encompasses three principal steps: constructing a scatter plot, calculating effect size and significance, and analyzing bottleneck levels. Initially, an X-Y scatter plot is generated, with upper boundary lines drawn based on data distribution. These upper boundary lines include Ordinary Least Squares (OLS), Convex Envelope-Free Disposal Hull (CE-FDH), and Convex Regression-Free Disposal Hull (CR-FDH). Above these boundary lines, a blank area emerges, whose magnitude represents the indispensability of X for Y. A larger blank area signifies a more substantial necessary condition effect. As illustrated in Figure 2, none of the condition variables demonstrate a necessary condition scale effect on public health governance capacity.

Through quantitative assessment, the necessity of individual antecedent conditions is evaluated by analyzing their effect sizes and statistical significance. A condition is considered necessary when its effect size (d) exceeds 0.1 and Monte Carlo permutation tests demonstrate statistical significance. The NCA method employs two estimation approaches: ceiling regression (CR) for continuous variables and ceiling envelopment (CE) for categorical variables.

Table 2 presents the NCA results for individual condition necessity, encompassing precision, ceiling area, range, effect size, and *p*-values derived from both CR and CE estimation methods. The analysis reveals that while doctor density, nursing and midwifery density, universal health coverage, and government health expenditure showed statistically significant results, their effect sizes are too small (*d* < 0.1) to constitute necessary conditions for public health governance capacity. PM_2.5_ concentration and tobacco use rates are neither statistically significant (*p* > 0.5) nor substantial enough (*d* < 0.1) to be considered a necessary condition. Consequently, no single antecedent condition independently generates high public health governance capacity.

**Table 2:** The NCA analysis results of the necessary conditions.

Antecedent variables	Method	Precision	Upper region	Range	Effect size d	p-value
Doctor density	CR	98.7%	0.016	1.00	0.016	0.000
	CE	100%	0.013	1.00	0.013	0.000
Nursing and midwifery density	CR	99.3%	0.000	1.00	0.000	0.004
	CE	100%	0.001	1.00	0.001	0.006
Universal health coverage	CR	92.6%	0.068	0.98	0.007	0.000
	CE	100%	0.014	0.98	0.015	0.000
Government health expenditure	CR	98.0%	0.003	0.99	0.003	0.000
	CE	100%	0.002	0.99	0.003	0.000
PM_2.5_ concentration	CR	100%	0.000	1.00	0.000	1.000
	CE	100%	0.000	1.00	0.000	1.000
Tobacco use rate	CR	100%	0.000	1.00	0.000	1.000
	CE	100%	0.000	1.00	0.000	1.000

CR indicates ceiling regression (an estimation approach for continuous variables in NCA); CE indicates ceiling envelopment (an estimation approach for categorical variables in NCA); effect size d indicates a metric measuring the magnitude of necessary condition effects, where 0 ≤ d < 0.1 indicates “low level”; 0.1 ≤ d < 0.3 indicates “moderate level”; p-value indicates the permutation test (resampling times = 10,000) in NCA analysis, where a p-value closer to 0 suggests a more statistically significant effect.

A further analysis explored the bottleneck levels. These levels represent the specific threshold within an antecedent condition’s maximum observed range that is necessary to achieve the result’s maximum observed scope. As illustrated in Table 3, the bottleneck level analysis reveals nuanced insights into public health governance capacity. To achieve 70% of public health governance capacity, a 5.7% universal health coverage is required. To achieve 80% of public health governance capacity, a 17.1% universal health coverage is required. To achieve 90% public health governance capacity, doctor density must be at least 5.5% and universal health coverage must reach 28.5%. At the maximum 100% public health governance capacity, doctor density must reach 24.0%, nursing and midwifery density 10.7%, universal health coverage 39.9%, and government health expenditure 20.6%. These findings underscore that higher levels of public health governance capacity are constrained by the simultaneous interaction of multiple conditions.

**Table 3:** The NCA analysis results of the bottleneck level (%).

Public health governance capacity	Doctor density	Nursing and midwifery density	Universal health coverage	Government health expenditure	PM_2.5_ concentration	Tobacco use rate
0	NN	NN	NN	NN	NN	NN
10	NN	NN	NN	NN	NN	NN
20	NN	NN	NN	NN	NN	NN
30	NN	NN	NN	NN	NN	NN
40	NN	NN	NN	NN	NN	NN
50	NN	NN	NN	NN	NN	NN
60	NN	NN	NN	NN	NN	NN
70	NN	NN	**5.7**	NN	NN	NN
80	NN	NN	**17.1**	NN	NN	NN
90	**5.5**	NN	**28.5**	NN	NN	NN
100	**24.0**	**10.7**	**39.9**	**20.6**	NN	NN

NN represents “not necessary,” and the bottleneck level analysis result was calculated using the CR method. The bold values denote the minimum level (%) of the corresponding antecedent condition required to achieve a specific level (%) of public health governance capacity.

#### 5.1.2 Analysis of the single necessary condition for fsQCA

I employed fsQCA to validate the robustness of the NCA results. Following Fiss (126), a condition is considered necessary for an outcome when its consistency level exceeds 0.9. Table 4 presents the necessity condition test results for high and low public health governance capacities. The analysis reveals that all conditions exhibit consistency levels below 0.9, thereby suggesting no necessary conditions exist for either high or low public health governance capacity. These findings are consistent with the NCA method’s previous results. The analysis demonstrates that public health governance capacity emerges from the complex interaction of multiple factors rather than being determined by a single condition, thus necessitating a configuration analysis.

**Table 4:** The necessity analysis of the QCA method.

Antecedent variables	High public health governance capacity		Low public health governance capacity	
	Consistency	Coverage	Consistency	Coverage
High doctor density	0.734	0.754	0.335	0.344
Low doctor density	0.361	0.352	0.760	0.741
High nursing and midwifery density	0.729	0.733	0.367	0.370
Low nursing and midwifery density	0.374	0.371	0.735	0.730
High universal health coverage	0.798	0.779	0.334	0.326
Low universal health coverage	0.310	0.318	0.774	0.793
High government health expenditure	0.681	0.685	0.427	0.429
Low government health expenditure	0.432	0.430	0.687	0.683
High PM_2.5_ concentration	0.645	0.628	0.494	0.481
Low PM_2.5_ concentration	0.466	0.480	0.617	0.635
High tobacco use rate	0.446	0.442	0.638	0.632
Low tobacco use rate	0.628	0.634	0.436	0.440

The necessity condition test for high/low public health governance capabilities using fsQCA shows that the consistency levels of all conditions are below 0.9, indicating that no single factor is a necessary condition for achieving high public health governance capabilities.

### 5.2 Configuration analysis

Configuration analysis represents a critical component of the fsQCA methodology. Utilizing fsQCA 3.0 software, this study examined the diverse configurational pathways associated with high public health governance capacity. The analytical process encompasses two primary analytical stages.

#### 5.2.1 Constructing truth table

Constructing the truth table requires establishing both case frequency and consistency thresholds. The case frequency threshold is primarily determined by sample size and the proportion of cases. Typically, for larger sample sizes, the case frequency threshold should exceed 1. In this study, I set the case frequency threshold at 2, with an initial consistency threshold of 0.8 and a Proportional Reduction in Inconsistency (PRI) level of 0.70. Consequently, only condition combinations appearing in at least two cases were included in the analysis, and only configurations with an original consistency above 0.8 were considered sufficient condition combinations potentially leading to high public health governance capacity.

#### 5.2.2 Identifying core and peripheral conditions

Drawing on existing public health governance theory, this study selected six critical antecedent conditions and employed counterfactual analysis to examine their differential impacts on public health governance capacity. Specifically, by comparing the nested relationships between intermediate and parsimonious solutions, the research identified core and peripheral conditions influencing public health governance capacity. Factors simultaneously present in both intermediate and parsimonious solutions were characterized as core conditions, while factors existing only in intermediate solutions but absent from parsimonious solutions were defined as peripheral conditions, thereby clarifying the relative importance of various conditions within configurations. The fsQCA analysis results are illustrated as shown in Table 5. In this table, large 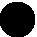
represents core condition presence, small 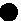 represents peripheral condition presence, large 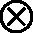 represents core condition absence, small 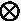 represents peripheral condition absence, and a blank indicates that condition may be present or absent.

**Table 5:** Configurations that produce high public health governance capacity.

Antecedent variables	Technological-Organizational				Comprehensive-driven	Organizational-driven	Organizational -Environmental
	C1	C2	C3	C4	C5	C6	C7
Doctor density	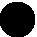	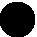	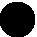	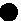	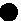	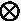	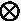
Nursing and midwifery density		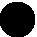	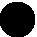	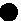	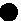	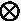	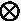
Universal health coverage	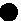	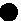	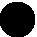	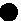	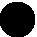	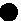	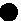
Government health expenditure	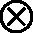	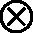		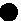	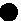	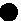	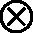
PM_2.5_ concentration	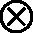	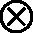	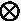		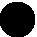	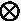	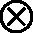
Tobacco use rate	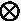		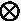	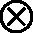		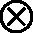	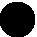
Consistency	0.892	0.901	0.869	0.836	0.838	0.885	0.874
Raw coverage	0.139	0.159	0.179	0.380	0.477	0.067	0.072
Unique coverage	0.007	0.021	0.003	0.006	0.131	0.014	0.016
Solution consistency	0.846						
Solution coverage	0.631						

Large 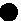 represents core condition presence; small 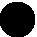 represents peripheral condition presence; large 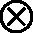 represents core condition absence; small 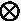 represents peripheral condition absence; blank indicates that condition may be present or absent.

Table 5 presents seven driving pathways for explaining high public health governance capacity. Each column represents a possible condition configuration. The solution consistency is 0.846, indicating that 84.6% of global cases meeting these seven condition configurations demonstrate high public health governance capacity. The solution coverage is 0.631, meaning that the seven condition configurations can explain 63.1% of high public health governance capacity cases. Both solution consistency and coverage exceed critical thresholds, validating the empirical analysis. Based on the condition configurations, the differentiated adaptive relationships among technological, organizational, and environmental factors in promoting public health governance capacity can be further identified. Based on the logical framework of antecedent conditions, this study categorizes the configurations that generate high public health governance capacity into four types: Technological-Organizational type, Comprehensive-driven type, Organizational-driven type, and Organizational-Environmental type.

The Technological-Organizational type refers to a pattern where technological and organizational factors interact to promote high public health governance capacity, specifically including four pathways C1, C2, C3, C4.

Configuration 1 (C1) indicates that universal health coverage plays a core role, with doctor density serving a complementary function. This pattern shows a raw coverage of 0.139 and unique coverage of 0.007, suggesting that this configuration explains 13.9% of cases with high-level public health governance capacity, while approximately 0.7% of public health governance capacity cases can only be explained by this particular pathway. Representative countries include China, Malaysia, Kazakhstan and Poland.

Configuration 2 (C2) demonstrates that universal health coverage serves as a core condition, with doctor density, nursing and midwifery density playing complementary roles. With a raw coverage of 0.159 and unique coverage of 0.021, this configuration accounts for 15.9% of cases with high-level public health governance capacity, while approximately 2.1% of public health governance capacity cases are uniquely explained by this pathway. Representative countries include Qatar, Kuwait, Kazakhstan, Poland and Turkmenistan.

Configuration 3 (C3) reveals that doctor density, nursing and midwifery density, and universal health coverage all function as complementary conditions. This configuration yields a raw coverage of 0.179 and unique coverage of 0.003, explaining 17.9% of cases with high-level public health governance capacity, while approximately 0.3% of public health governance capacity cases can only be explained through this pathway. Representative countries include Australia, Norway, Sweden, Uruguay, Denmark, Iceland, Germany, Netherlands, New zealand, United States, Austria, Portugal, Finland, France, Malta, UK, Spain and Russian Federation.

Configuration 4 (C4) demonstrates that doctor density, nursing and midwifery density, universal health coverage, and government health expenditure all serve as core conditions. With a raw coverage of 0.380 and unique coverage of 0.006, this configuration explains 38.0% of cases with high-level public health governance capacity, while approximately 0.6% of public health governance capacity cases are uniquely explained by this pathway. Representative countries include Thailand and Algeria.

The Comprehensive-driven type refers to a pattern where technological, organizational, and environmental factors interact to promote high public health governance capacity, specifically including pathway C5.

Configuration 5 (C5) demonstrates that doctor density, nursing and midwifery density, and government health expenditure all serve as core conditions, while universal health coverage and PM_2.5_ concentration function as complementary factors. With a raw coverage of 0.477 and unique coverage of 0.131, this configuration explains 47.7% of cases with high-level public health governance capacity, while approximately 13.1% of public health governance capacity cases can only be explained through this specific pathway. The sole representative country is Bahrain.

The Organizational-driven type refers to a pattern that relies exclusively on organizational factors to promote high public health governance capacity, specifically including pathway C6.

Configuration 6 (C6) demonstrates that universal health coverage and government health expenditure both function as core conditions. This configuration indicates that high-level public health governance capacity can be achieved through strong universal health coverage and substantial government health expenditure, even without significant technological or environmental factors. With a raw coverage of 0.067 and unique coverage of 0.014, this configuration explains 6.7% of cases with high-level public health governance capacity, while approximately 1.4% of public health governance capacity cases can only be explained through this specific pathway. Representative countries include Kazakhstan, Chile, Poland, Republic of Korea, Israel and Bulgaria.

The Organizational-Environmental type refers to a pattern where organizational and environmental factors interact to promote high public health governance capacity, specifically including pathway C7.

Configuration 7 (C7) reveals that universal health coverage serves as a core condition, while tobacco use rate functions as a complementary factor. This configuration demonstrates that high-level public health governance capacity can be achieved with strong universal health coverage alongside high tobacco use rates, even without considering other technological factors. With a raw coverage of 0.072 and unique coverage of 0.016, this configuration explains 7.2% of cases with high-level public health governance capacity, while approximately 1.6% of public health governance capacity cases can only be explained through this specific pathway. Representative countries include Czechia, Austria, France, Portugal, Spain, Malta, Russian Federation, Sweden, Estonia, Italy, United States, Lithuania, Netherlands, Belarus, Germany, Slovenia and Chile.

The findings show that among the seven pathways to enhance national public health governance capacity, representative countries are predominantly high-income, with some upper-middle-income countries included. Only Algeria from lower-middle-income countries is represented, as its government-funded public healthcare system provides free services to all citizens, covering 90% of the national population. Notably, no low-income countries appear in any pathway. This overall absence of lower-middle- and low-income countries, with only rare exceptions, is not accidental but rather the result of interconnected multifaceted factors.

Among the original dataset of 194 countries, the sample comprised 59 high-income countries, 59 upper-middle-income countries, 48 lower-middle-income countries, and 28 low-income countries. Of these, 45 countries had missing data: 12 high-income, 14 upper-middle-income, 13 lower-middle-income, and 6 low-income countries. This distribution suggests a relatively balanced pattern of data missingness across income groups. Data gaps were influenced by random factors such as national statistical capacities and data transparency. For instance, some high-income countries might withhold specific healthcare expenditure details due to privacy regulations, while certain lower-middle-income countries experience data discontinuity because of armed conflicts or incomplete statistical systems. Critically, all indicators were sourced from the WHO’s standardized database (World Health Statistics 2023), ensuring consistent definitional and statistical standards across countries and minimizing cross-national measurement bias. Consequently, the underrepresentation of lower-middle- and low-income countries in high-governance pathways is not attributable to data missingness or analytical bias. Instead, it reflects the structural constraints confronting their public health systems, which impede the formation of the requisite condition combinations for these pathways.

From an economic perspective, lower-middle- and low-income countries consistently face fiscal resource constraints. The seven identified pathways rely on synergistic interactions among technological, organizational, and environmental factors, each requiring stable resource investments. For instance, technical conditions such as doctor density and nursing and midwifery density demand long-term medical education and salary guarantees. Organizational conditions like UHC and government health expenditure depend on sustained fiscal support. Environmental conditions, including PM_2.5_ governance and tobacco control, necessitate inter-sectoral institutional collaboration costs. High-income countries leverage their substantial GDP and fiscal revenues to simultaneously meet multidimensional resource requirements. Conversely, some low-income countries’ per capita GDP is less than one-tenth of high-income nations. Their limited fiscal resources are primarily allocated to addressing survival-level crises such as famine and armed conflicts, rendering systematic public health system development challenging (60, 62). According to the WHO’s latest report, “Global Spending on Health: Emerging from the Pandemic,” high-income countries’ per capita healthcare expenditure reaches $3,731. This is 28 times the amount in lower-middle-income countries ($132) and 87 times that in low-income countries ($43). Such gaps directly prevent them from meeting core conditions in the pathways, such as “high government health expenditure” and “high UHC.”

From a resource allocation perspective, political leadership’s attention and prioritization critically shape public investment trajectories (127). Lower-middle- and low-income countries have long confronted persistent challenges from endemic diseases like malaria and cholera. Consequently, their limited resources are predominantly channeled into short-term emergency interventions, such as vaccination campaigns and disease containment, rather than long-term systemic capacity building, including enhanced monitoring systems and cross-sectoral collaboration mechanisms. In this study, high-governance pathways predominantly feature preventive governance strategies. In contrast, lower-middle- and low-income countries remain predominantly reactive, focused on immediate response rather than proactive management. This fundamental priority divergence inhibits their ability to meet the pathways’ complex requirements for synchronized technological, organizational, and environmental factors (61).

From an institutional standpoint, governance structures in lower-middle- and low-income countries struggle to support synergistic mechanisms within these pathways. Research identifies UHC is present across all pathways, and its implementation requires a stable political environment and efficient administrative capacity. Lower-middle- and low-income countries commonly face issues like political instability and low administrative efficiency. Even when UHC goals are established, they rarely translate into tangible governance effectiveness (128).

From the perspective of international assistance limitations, external support has failed to bridge the capacity gaps in lower-middle- and low-income countries. Existing international assistance predominantly concentrates on vertical interventions targeting specific diseases, such as HIV/AIDS and malaria, rather than systematic governance capacity building (129). Moreover, amid rising global uncertainties and intensifying fiscal resource competition, health development assistance has entered a period of significant contraction (130).

In summary, the absence of lower-middle- and low-income countries from optimal health pathways stems from a complex interplay of economic resource constraints, prioritization disparities, institutional environments, and international assistance models. This phenomenon underscores that the pathways identified in this research are most applicable to countries with robust economic foundations and governance resilience. Consequently, lower-middle- and low-income countries must explore differentiated approaches aligned with their unique resource endowments. Ultimately, this analysis reveals profound inequalities inherent in global public health governance.

### 5.3 Robustness test

To assess the robustness of the configuration analysis, robustness tests are conducted by adjusting the consistency threshold and the PRI consistency level. Reducing the consistency threshold from 0.80 to 0.75 produced no changes in the overall configuration distribution, core conditions, or boundary combinations. When the PRI consistency level is increased from 0.70 to 0.75, the resulting new configurations could be viewed as a subset of the original configurational pathways, with configuration 4 being eliminated while the remaining six pathways converge with pre-test results. These findings demonstrate the robustness of the configuration analysis results.

In discussing external validity in large-sample QCA research, Rutten (131) conducted ten repeated analyses by randomly removing 10 cases, comparing the frequency and status of original configurational pathways across these analyses to calculate precision. Following this approach, 10 cases were randomly removed from the sample of 149 countries, followed by truth table reanalysis repeated ten times. Panel A of Table 6 presents these results. C1 is fully replicated in five of the ten reanalyses, identified as its superset (a broader combination of conditions, meaning the original configurational pathway is a subset of the new configurational pathway) in one instance, and remains unidentified in four instances. The precision of C1 is (5 + 1)/10 = 0.6. C6 is fully replicated in six of the ten reanalyses, identified as a subset (a narrower combination of conditions, meaning the new configurational pathway is a subset of the original configurational pathway) in one instance, and remains unidentified in three instances. The precision of C6 is (6 + 1)/10 = 0.7. Following the same calculation method, C2, C3, C4, C5, and C7 demonstrate precision scores of 1.0, 0.6, 0.8, 1.0, and 0.8, respectively. The overall precision across all configurations is 0.79.

**Table 6:** Robustness test of case deletion.

Panel A: Accuracy analysis with 10 cases deleted								
Identified as	C1	C2	C3	C4	C5	C6	C7	Accuracy
●	5 times	8 times	6 times	8 times	10 times	6 times	8 times	51
◓	1 times	2 times						3/70 = 0.04
◒						1 times		1/70 = 0.01
⊗	4 times		4 times	2 times		3 times	2 times	15/70 = 0.21
Accuracy	6/10 = 0.60	10/10 = 1.00	6/10 = 0.60	8/10 = 0.80	10/10 = 1.00	7/10 = 0.70	8/10 = 0.80	(6 + 10 + 6 + 8 + 10 + 7 + 8) /70 = 0.79

Panel B: Accuracy analysis with 10% of cases deleted								
Identified as	C1	C2	C3	C4	C5	C6	C7	Accuracy
●	6 times	6 times	6 times	8 times	8 times	5 times	6 times	45
◓	2 times	2 times						4/70 = 0.06
◒	1 times	1 times			2 times	1 times		5/70 = 0.07
⊗	1 times	1 times	4 times	2 times		4 times	4 times	16/70 = 0.23
Accuracy	9/10 = 0.90	9/10 = 0.90	6/10 = 0.60	8/10 = 0.80	10/10 = 1.00	6/10 = 0.60	8/10 = 0.80	(9 + 9 + 6 + 8 + 10 + 6 + 6) /70 = 0.77

● replicated ◓ identified as superset ◒identified as subset ⊗ not identified; C1-C4 are technology-organizational type, C5 is comprehensively driven type, C6 is organizationally driven type, and C7 is organizational-environmental type.

Following Rutten’s (131) methodology, who randomly removed 10 cases (approximately 10%) from a sample of 108, this study randomly removed 10% of the 149 cases-15 countries-and performed a new truth table analysis. This process was repeated 10 times, with results presented in Panel B of Table 6. C1, C2, C3, C4, C5, C6 and C7 demonstrate precision scores of 0.9, 0.9, 0.6, 0.8, 1.0, 0.6, and 0.6, respectively. The overall precision across all configurations is 0.77. These results indicate that the configurational solutions identified in this study remain generally robust when experimental conditions are varied.

## 6 Conclusion and policy implications

### 6.1 Conclusion

Public health emergencies consistently challenge governmental public health governance capacity. Effectively responding to these events remains a critical concern for both government decision-makers and academia. This study analyzes public health governance capacity across 149 countries using NCA and fsQCA to test and extend the TOE framework in a global context, thereby exploring how technological, organizational, and environmental conditions configure to drive public health governance capacity. This study reveals that: (1) The NCA results reveal that no single condition variable constitutes a necessary condition for high-level or low-level public health governance capacity. (2) The fsQCA results reveal that high-level public health governance capacity emerges from various combinations of technological, organizational, and environmental condition variables, manifesting in seven distinct pathways that collectively drive capacity enhancement. These include four technological-organizational pathways, one comprehensive-driven pathway, one organizational-driven pathway, and one organizational-environmental pathway. Notably, the comprehensive-driven pathway plays a more significant role in improving public health governance capacity, indicating that the synergy among different conditions is crucial. (3) Among these configurations, universal health coverage demonstrates a relatively universal effect in enhancing public health governance capacity, followed by doctor density and nursing and midwifery density. (4) The representative countries across the seven pathways predominantly consist of high-income nations, with some upper-middle-income countries also represented. However, lower-middle-income and low-income countries are virtually absent.

This study provides new perspectives and analytical tools for theoretical development in the field of public health. Firstly, by integrating the Resource-Based View (RBV), Institutional Theory, and Social Determinants of Health (SDOH) theory, it constructs a multi-level, interdisciplinary TOE research framework. This framework organically combines technological, organizational, and environmental dimensions, systematically explaining the core factors influencing public health governance capacity and their interactive relationships. Transcending the limitations of previous studies that often focused on a single theory or dimension, this framework not only enriches the conceptual landscape of public health governance but also offers a new analytical perspective for understanding governance mechanisms within complex social systems. Secondly, this study breaks through the constraints of traditional linear analysis frameworks by introducing the configurational perspective into research on national-level public health governance capacity. The findings reveal the phenomenon of “different routes leading to the same destination” in the pathways for improving public health governance capacity across various countries. This means that different countries can achieve similar governance performance through differentiated combinations of factors, a discovery that transcends the simplistic understanding of “best practices” in traditional research and provides a more nuanced and reality-aligned theoretical lens for interpreting differences in governance capacity among countries.

### 6.2 Policy implications

Based on the findings, enhancing public health governance capacity depends on the synergistic configuration of technological, organizational, and environmental factors. Such synergistic effects are most pronounced in upper-middle-income and higher-income countries, particularly high-income ones. Thus, targeted strategies should be developed according to differences in resource endowments across countries with varying income levels.

Lower-middle- and low-income countries have few representative cases in high public health governance pathways, with only Algeria serving as a representative of lower-middle-income countries. This suggests that such countries face more severe public health governance challenges. They also suffer from comprehensive shortages in human resources and financial capacity, making it difficult to directly replicate the successful pathways of high-income countries.

Algeria’s experience demonstrates that even with constrained resources, a government-led healthcare system can provide essential medical services to the majority of the population. Consequently, lower-middle- and low-income countries should prioritize allocating limited resources to preventive health services and basic medical coverage. By establishing a network of community health centers, these countries can ensure basic medical services reach the broadest population segments. Moreover, governments must leverage legislative and institutional design to maintain a stable healthcare expenditure proportion within national budgets, thus preventing significant reductions in health investments during economic fluctuations. In human resource development, emphasis should be placed on training primary healthcare workers. Given resource constraints, these countries can scale up community health worker programs, which deliver basic medical and preventive care to rural and remote areas through short-term training. Additionally, collaborations with international organizations and high-income countries should be established to develop medical education and training initiatives, boosting the professional competence of local healthcare personnel. In terms of environmental sanitation, priority should be given to developing basic sanitation infrastructure, such as safe drinking water supplies, solid waste disposal, and basic sewage systems. These infrastructures are critical for preventing the spread of infectious diseases. Additionally, efforts should strengthen environmental monitoring capacities and establish simple yet effective systems for environmental health risk assessment and early warning. In terms of international assistance, countries should proactively seek international assistance and technical support, promoting a shift in international assistance from single disease control programs to comprehensive health system capacity building. Additionally, effective international assistance management mechanisms should be established to ensure that assistance funds and resources maximize their impact, avoiding resource waste and duplicative efforts. Finally, these countries should strengthen South–South cooperation, share experiences and resources with other developing countries, and jointly address public health challenges.

Representative cases of upper-middle-income countries in public health governance pathways include China, Malaysia, Kazakhstan, Turkmenistan, Thailand, and Belarus, spanning technological-organizational, organizational-driven, and organizational-environmental pathways. High-income countries have the most representative cases in public health governance pathways, distributed across various approaches. This indicates that both upper-middle-income and high-income countries can adopt multiple pathways to strengthen public health governance capacity. For upper-middle-income and high-income countries not yet represented as case examples, they should draw on the successful experiences of representative countries. First, these countries need to assess their current status across the three dimensions of technology, organization, and environment. Through benchmarking against representative countries, they should clarify their current development stage, identify their strengths and weaknesses, and subsequently select the most suitable models for learning and pathway approaches. And the learning process should fully consider their own historical traditions, cultural backgrounds, social systems, and resource endowments, adopting a gradual, step-by-step approach. Finally, learning assessment mechanisms should be established to regularly evaluate learning effectiveness. Key performance indicators (KPIs) should be set to monitor improvements in public health governance capacity. Moreover, attention should be paid to the latest developments in representative countries to promptly adopt new experiences and practices, ensuring the learning process remains dynamically updated. For representative case countries, despite possessing relatively advanced public health governance capabilities, emerging challenges continually reshape the landscape. Key issues such as population aging, increasing chronic disease burdens, and emerging infectious diseases demand ongoing adaptation of public health governance systems. Furthermore, countries representing different governance pathways must proactively identify and mitigate pathway-specific risks to develop more balanced and resilient public health governance systems.

High-income countries should assume greater international responsibilities commensurate with their capabilities and strengthen the global health governance system. As countries with relatively abundant public health resources, they should provide more technical assistance and resource support to help low- and middle-income countries and least developed countries build core public health capacities. Specifically, through multilateral or bilateral cooperation mechanisms, they can offer support in areas such as training for public health personnel, technology transfer, and infrastructure development in developing countries. In the context of globalization, emerging public health threats, including emerging infectious diseases, antimicrobial resistance, and the health impacts of climate change, are becoming increasingly prominent. High-income countries should strengthen international cooperation and coordination to collectively address global public health challenges.

### 6.3 Limitations and future research directions

Although this study analyzed the factors influencing public health governance capacity in 149 countries, there are still some limitations in the research process. Meanwhile, these limitations also point to directions for in-depth exploration in subsequent research.

First, while this study employed NCA and fsQCA to explore the necessity and configuration effects of different conditions, these methods have inherent limitations. The NCA method relies on data distribution characteristics, which constrains precise identification and in-depth investigation of necessary relationships. Although fsQCA can identify multiple pathways leading to specific outcomes, it is sensitive to the setting of consistency thresholds, case frequencies, and PRI consistency levels. Even though robustness tests with adjusted parameters confirmed the stability of primary pathways, such subjective judgment remains a methodological constraint. Additionally, public health governance is a complex social system. Beyond the technical, organizational, and environmental conditions examined in this study, it is influenced by deeper factors such as political systems, cultural values, and social trust. Due to limitations in data availability, this study could not incorporate these potentially important variables into its analytical framework. However, further increasing the number of factors would risk excessive fragmentation of configurations. This would make it hard to identify explanatory core pathways, even leading to extreme cases where each case corresponds to a unique configuration. Such outcomes contradict fsQCA’s original aim of identifying common configurational patterns, which is another limitation of the method.

Second, this study primarily relies on the World Health Statistics 2023 dataset published by the WHO. While this data is authoritative, it depends on countries’ independent collection and reporting, giving rise to unavoidable statistical issues. For instance, Low-income countries often lack basic capacity to collect health data. Some regions still rely on paper records or manual aggregation, which can lead to underreporting, delayed submission, or incomplete records in remote areas. Moreover, the assessment of some core IHR capacity indicators involves a certain degree of subjectivity. These factors directly undermine the accuracy of core indicators and may compromise the validity of cross-country comparisons. Additionally, this study uses only cross-sectional data for analysis and cannot capture the dynamic evolution of public health governance capacity across countries over time. Improving public health governance capacity is a dynamic process. Iterations in technological progress, adjustments to organizational structures, shifts in environmental pressures, and other factors may alter configurational pathways. Short-term data, however, cannot reveal such long-term dynamic mechanisms nor verify the stability of core conditions across periods.

Third, within the new landscape of global public health governance, innovative technologies, including digital tools and artificial intelligence, are profoundly reshaping public health practices. While this study focuses on traditional factors in public health governance, it insufficiently explores the potential impacts of emerging technologies. Notably, in the wake of the COVID-19 pandemic, the global public health governance system is undergoing profound transformations. These developments offer rich avenues for future research.

These limitations do not diminish the scholarly value of this study. Instead, they provide directions for improvement in subsequent research. Future research could expand the theoretical framework by incorporating variables from other dimensions, refine research methods, and diversify data sources. Such efforts would further deepen understanding of the factors influencing public health governance capacity and their mechanisms of action. And this would offer a stronger theoretical foundation and practical guidance for building a more effective, equitable, and sustainable global public health governance system.

## Data Availability

The datasets presented in this study can be found in online repositories. The names of the repository/repositories and accession number(s) can be found below: https://figshare.com/s/19dfdb71a629fa633fab.
